# Identification of Novel Bromodomain-Containing Protein 4 (BRD4) Binders through 3D Pharmacophore-Based Repositioning Screening Campaign

**DOI:** 10.3390/molecules29174025

**Published:** 2024-08-26

**Authors:** Ester Colarusso, Erica Gazzillo, Eleonora Boccia, Stefania Terracciano, Ines Bruno, Giuseppe Bifulco, Maria Giovanna Chini, Gianluigi Lauro

**Affiliations:** 1 Department of Pharmacy, University of Salerno, Via Giovanni Paolo II 132, 84084 Fisciano, Italy; ecolarusso@unisa.it (E.C.); egazzillo@unisa.it (E.G.); eboccia@unisa.it (E.B.); sterracciano@unisa.it (S.T.); brunoin@unisa.it (I.B.); bifulco@unisa.it (G.B.); 2 Department of Biosciences and Territory, University of Molise, Contrada Fonte Lappone, 86090 Pesche, Italy

**Keywords:** drug repositioning, bromodomain, drug discovery, computational techniques, chemical synthesis, anticancer agents

## Abstract

A 3D structure-based pharmacophore model built for bromodomain-containing protein 4 (BRD4) is reported here, specifically developed for investigating and identifying the key structural features of the (+)-JQ1 known inhibitor within the BRD4 binding site. Using this pharmacophore model, 273 synthesized and purchased compounds previously considered for other targets but yielding poor results were screened in a drug repositioning campaign. Subsequently, only six compounds showed potential as BRD4 binders and were subjected to further biophysical and biochemical assays. Compounds **2**, **5**, and **6** showed high affinity for BRD4, with IC_50_ values of 0.60 ± 0.25 µM, 3.46 ± 1.22 µM, and 4.66 ± 0.52 µM, respectively. Additionally, these compounds were tested against two other bromodomains, BRD3 and BRD9, and two of them showed high selectivity for BRD4. The reported 3D structure-based pharmacophore model proves to be a straightforward and useful tool for selecting novel BRD4 ligands.

## 1. Introduction

Bromodomain-containing protein 4 (BRD4) is a member of the Bromodomain and Extra-Terminal (BET) family, which plays a pivotal role in regulating gene expression through its interaction with acetylated lysine residues on histone tails. BRD4 is critically involved in various cellular processes, including transcriptional elongation, DNA damage repair, cell cycle progression, cell proliferation, and apoptosis [1], making it a key target for therapeutic intervention in several cancers and inflammatory diseases. Specifically, BRD4 stands out in the BET family members as a particularly specific target for cancers such as Burkitt’s lymphoma, multiple myeloma (MM), and acute myeloid leukemia (AML) [2]. 

BRD4 binders compete with acetylated histones inhibiting their interaction onto the hydrophobic cavity in the protein binding site. This prevents the preferential recruitment of BRD4 to the promoter of target genes, leading to transcriptional suppression. Consequently, disrupting BRD4 function through small molecule inhibitors has emerged as a promising therapeutic strategy for identifying new anticancer agents [3,4]. However, due to the toxicity associated with first-generation inhibitors, which are often non-selective, there is an urgent need to select and develop new selective BRD4 inhibitors [2]. Therefore, the identification and optimization of novel BRD4 inhibitors with improved efficacy and selectivity remain a major focus of current research.

In this scenario, computational techniques have revolutionized the drug discovery process, enabling the rapid identification and optimization of potential drug candidates. In this study, we developed a 3D structure-based pharmacophore model [5,6] specifically built for accelerating the identification of BRD4 inhibitors, based on the chemical information from the crystal structure of this bromodomain-containing protein with (+)-JQ1 [7], which is a well-known potent BET inhibitor featuring potent anti-proliferative effects in multiple cancer models by binding BRD4 and preventing its interaction with chromatin.

The implementation of the developed pharmacophore model in a drug repositioning campaign represented a convenient strategy [8], enabling the recovering of molecules originally designed and synthesized for specific proteins, which failed to yield positive biological results. In this work, we report on the building and successful use of a BRD4-specific pharmacophore model integrated into an in silico drug repositioning campaign, which led to the identification of several novel promising BRD4 binders. 

The binding of these candidate molecules was subsequently validated through AlphaScreen assays, corroborating the predicted binding and the significant promise of the identified molecules as therapeutic agents targeting BRD4. To verify their selectivity against BRD4, we also tested the selected compounds on two other bromodomains [BRD3(BD1) and BRD9], obtaining promising outcomes.

Therefore, this study highlights the power of computational techniques in drug discovery, providing a robust framework for the development and repositioning of new BRD4 inhibitors. Our findings offer valuable insights into the structural and chemical determinants of BRD4 inhibition, paving the way for the development of more effective and selective therapeutic agents.

## 2. Results and Discussion

**Figure 1:**
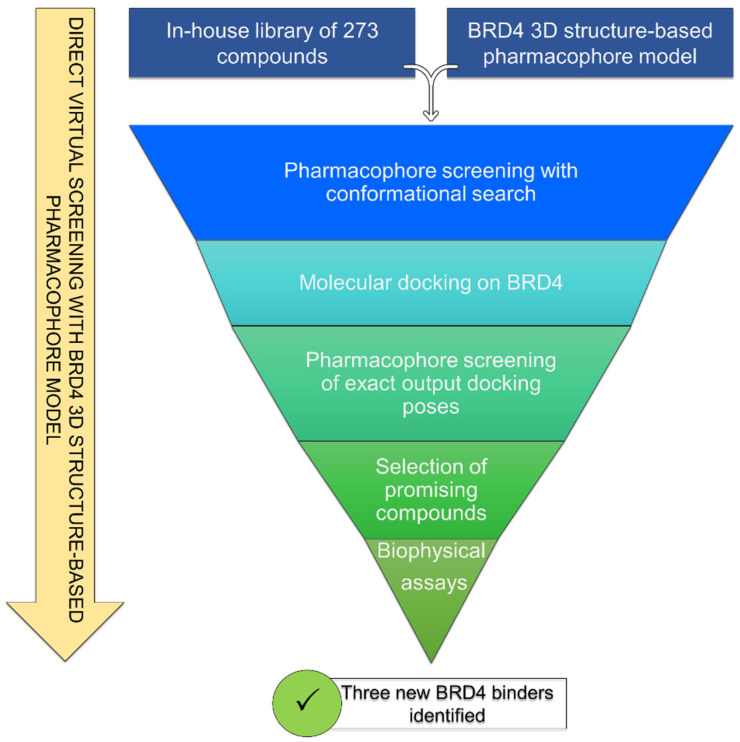
General workflow applied.

In this study, an in silico approach was applied for a drug repositioning campaign, considering an in-house library of 273 organic compounds (Table S1, Supporting Information), synthesized and purchased during the last few years and originally selected for different proteins of our interest (e.g., mPGES-1, HSP90, BRD9, TANK1, HSF1, BAG3). The computational repositioning campaign reported here is based on an in silico/experimental workflow (Figure 1), which led to the identification of three new promising items for BRD4. 

### 2.1. Development of 3D Structure-Based Pharmacophore Model of BRD4

**Figure 2:**
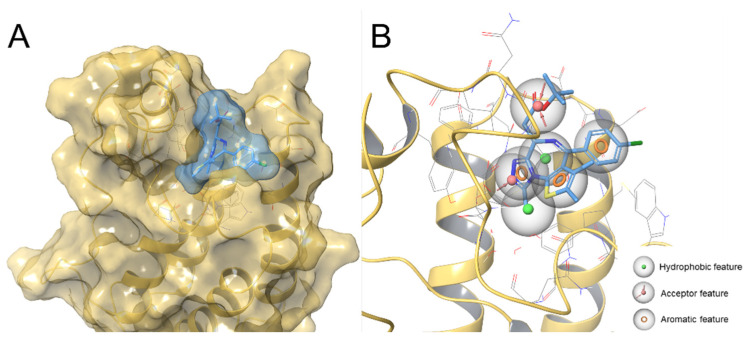
Panel (**A**) (+)-JQ1 (highlighted in light blue surface and colored by atom type: C light blue, O red, N blue, S yellow) in BRD4 (highlighted in light yellow surface) binding site (PDB code: 3MXF). Panel (**B**) Developed BRD4 structure-based pharmacophore model in BRD4 binding site (PDB code: 4QB3) superimposed onto (+)-JQ1.

The first step of this workflow concerned the development of a 3D structure-based pharmacophore model specific for BRD4. In particular, we selected the co-crystallized pose of (+)-JQ1 (PDB code: 3MXF [7]) as a reference structure for the development of the pharmacophore model. (+)-JQ1 was chosen as a representative potent BRD4 inhibitor featuring marked anticancer effects in different cancer types and featuring an excellent shape complementarity with the acetyl-lysine binding cavity of BRD4 (Figure 2A). Based on this evidence, the goal was to obtain a pharmacophore model that covered the main chemical features and reproduced the three-dimensional arrangement of (+)-JQ1 in the acetyl-lysine binding site of BRD4, in order to rapidly select compounds capable of binding the protein in a (+)-JQ1-like manner. With these premises, the crystal structure of the first bromodomain (BD1) of BRD4 co-crystallized with (+)-JQ1 (PDB code: 3MXF [7]) was superimposed onto the BRD4(BD1) crystal structure used for the subsequent in silico step, chosen for its high resolution (0.94 Å) (PDB code: 4QB3 [9]). In this way, the reference ligand (+)-JQ1 was put in the same system coordinates of the protein structure used for the molecular docking calculations, thus allowing for the generation of the pharmacophore model directly onto the protein coordinates subsequently considered for the structure-based virtual screening campaign. In detail, taking into account the chemical groups of (+)-JQ1, a seven-point model was obtained (Figure 2B and Computational Details), which contains the following: an acceptor “A1” and a hydrophobic “H1” feature (Figure S1), representative of the acetyl-lysine mimetic function and responsible for BRD4 binding and the establishment of the H bond with the fundamental amino acid Asn140; three aromatic features “R1”, “R2”, and “R3” (Figure S1); and one additional hydrophobic feature ”H2” (Figure S1), which was selected and considered in order to obtain the three-dimensional arrangement necessary for reproducing the shape complementarity and hydrophobic interaction patterns of the reference inhibitor. Moreover, a further acceptor group, namely “A2” (Figure S1), was selected to reproduce additional polar interactions (e.g., with Asn140), also considering the presence of an acceptor group in this position [10,11,12,13,14,15,16,17]. 

### 2.2. Drug Repositioning Campaign of In-House Library through Developed BRD4 3D Structure-Based Pharmacophore Model

**Figure 3:**
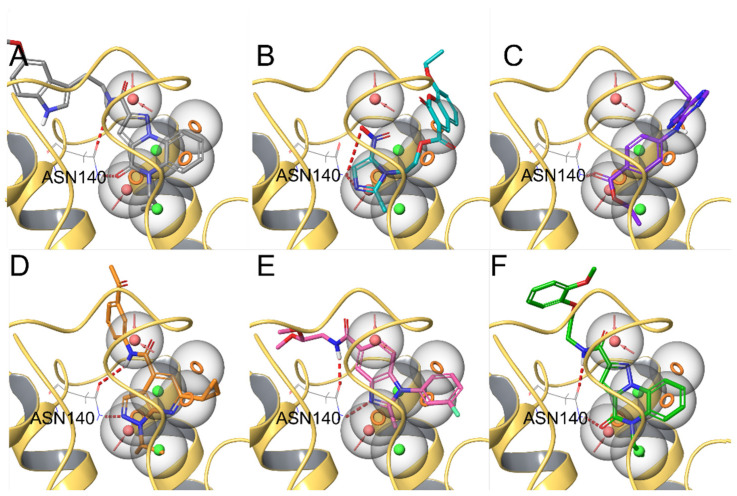
Compounds **1** (panel (**A**), colored by atom type: C gray, O red, N blue, polar H light gray), **2** (panel (**B**), colored by atom type: C turquoise, O red, N blue, polar H light gray), **3** (panel (**C**), colored by atom type: C violet, O red, N blue, polar H light gray), **4** (panel (**D**), colored by atom type: C orange, O red, N blue, polar H light gray), **5** (panel (**E**), colored by atom type: C pink, O red, N blue, polar H light gray, Cl light green), and **6** (panel (**F**), colored by atom type: C green, O red, N blue, polar H light gray) docking poses in BRD4 binding site (PDB code: 4QB3) superimposed onto developed BRD4 pharmacophore model. H bonds are highlighted in red.

The developed BRD4 pharmacophore model was employed for a drug repositioning campaign, considering an in-house library of 273 synthesized and purchased compounds, originally selected for different proteins. Specifically, applying the workflow reported in Figure 1, these compounds were subjected to a first round of ligand-based pharmacophore screening, namely performing a conformational search for each compound in order to sample the related geometries and to exclude the items that a priori did not respect the pharmacophoric features. Specifically, we set 5/7 matches as the minimum number of features to respect, considering the fact that the developed BRD4 pharmacophore represented a highly selective filter. Indeed, it was generated based on the chemical information of (+)-JQ1, which represents an excellent binder of BRD4 due to its ability to establish an extensive network of interactions with the protein counterpart [7]. Applying this preliminary filter, among the starting 273 compounds, 158 items showed the respect of 5/7 pharmacophoric points and were then subjected to the subsequent structure-based molecular docking calculations. After this key step, the obtained docking poses were further submitted to a very restrictive screening round, namely considering structure-based pharmacophore screening, which represented the core of the entire screening. In fact, differently from the first round of pharmacophore screening, namely ligand-based pharmacophore screening (Figure 1), in this case, we considered the exact docking pose of the ligand in the BRD4 binding site, thus accounting for the binding conformation of the molecule onto the binding pocket of the protein and thus introducing a restrictive filter in the workflow (Figure 1). As expected, only six compounds (**1**–**6**, Figure 3 and Figure S2, Table 1) respected 5/7 matches of the pharmacophore model and were then selected for the subsequent biological evaluation to assess their predicted ability to act as new promising BRD4 binders. 

### 2.3. Evaluation of In Silico Predicted Binding on BRD4 by AlphaScreen Assay

**Figure 4:**
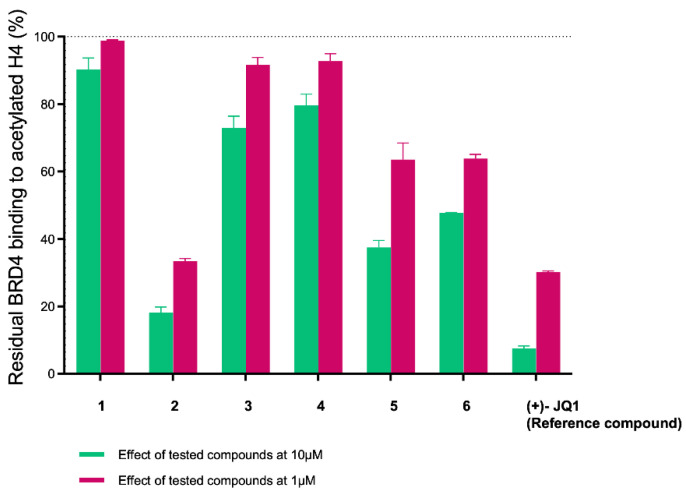
Residual binding of histone H4Ac ± SD (%) to BRD4(BD1) after treatment with compounds **1**–**6** at 10 and 1 μM.

To validate the entire in silico pipeline and demonstrate its potential uses in drug discovery, we selected the AlphaScreen technique as a suitable assay for this purpose. Compared to other binding assays, AlphaScreen is a competitive assay that evaluates the ability of new compounds to displace the natural binder (histone H4/Ac for BRD4) from the BRD binding site [18]. In light of this, we tested the selected compounds **1**–**6** (Table 1) against BRD4(BD1) at concentrations of 10 µM and 1 µM (Figure 4). Interestingly, compounds **2**, **5**, and **6** demonstrated the ability to bind the target protein at both concentrations (residual binding of histone H4Ac: 18.2 ± 2.9% to 47.7 ± 0.3% at 10 µM and 33.5 ± 0.7% to 63.5 ± 4.9% at 1 µM). Compounds **3** and **4** showed only weak binding at 10 µM (residual binding of histone H4Ac: 72.9 ± 4.8% and 79.6 ± 4.7%, respectively), while compound **1** was the least active, with a residual binding of histone H4Ac at 90.3 ± 4.8% (Figure 4 and Table 2).

Thereafter, the half-maximal inhibitory concentration (IC_50_) values of the most effective compounds were determined, confirming that all could bind to the BRD4 protein in the low micromolar range.

**Figure 5:**
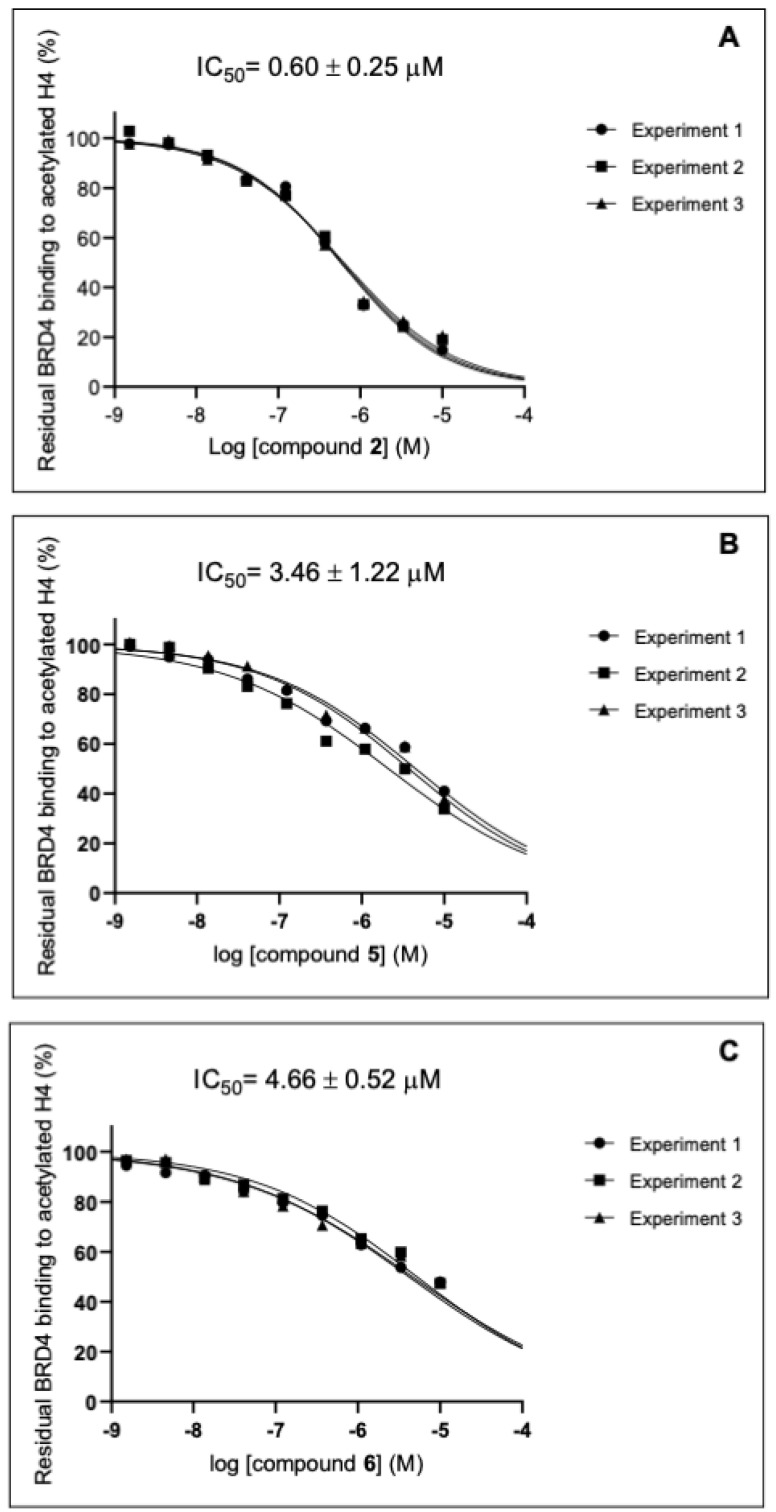
IC_50_ curves of compounds **2** (**A**), **5** (**B**), and **6** (**C**) on BRD4(BD1). Data are presented as means with standard deviation (SD), with *n* = 3.

Specifically, the IC_50_ values were found to be 0.60 ± 0.25 µM for **2**, 3.46 ± 1.22 µM for **5**, and 4.66 ± 0.52 µM for **6** (Figure 5 and Table 2). The low micromolar IC_50_ values indicate that these compounds have a strong affinity for the BRD4 binding site, supporting their potential for further development in drug discovery efforts. These results confirm the potency of computational outcomes in identifying potential drug candidates capable of binding BRD4 and the effectiveness of the AlphaScreen assay as the most appropriate method to confirm these findings.

### 2.4. Evaluation of Selectivity among Other Bromodomains

A great deal of clinical research on BET inhibitors has validated BRD4 as a therapeutic target for cancer and other diseases, and numerous BRD4 inhibitors have reached the clinical phase (e.g., (+)-JQ1, GSK2820151, I-BET151) [2,19]. However, these studies have highlighted several reversible side effects attributed to the non-selective nature of these bromodomain inhibitors, also known as pan-BET inhibitors [2,19]. 

**Figure 6:**
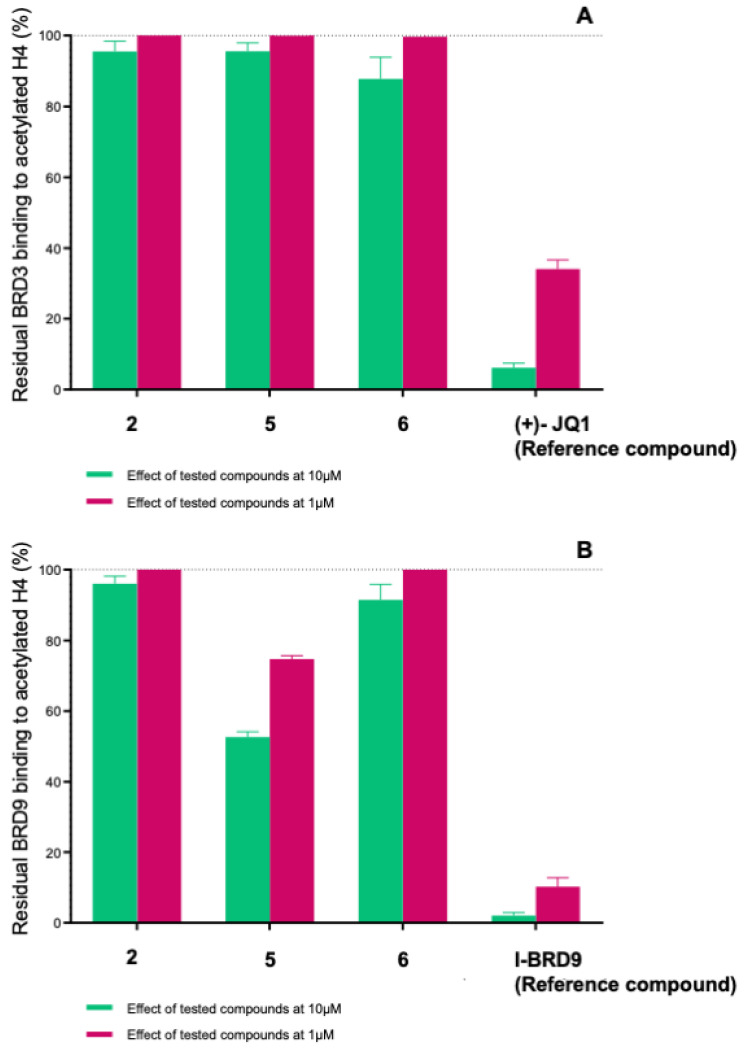
Residual binding of histone H4Ac ± SD (%) to BRD3(BD1) (**A**) and BRD9 (**B**) after treatment with compounds **2**, **5**, and **6** at 10 and 1 μM.

**Figure 7:**
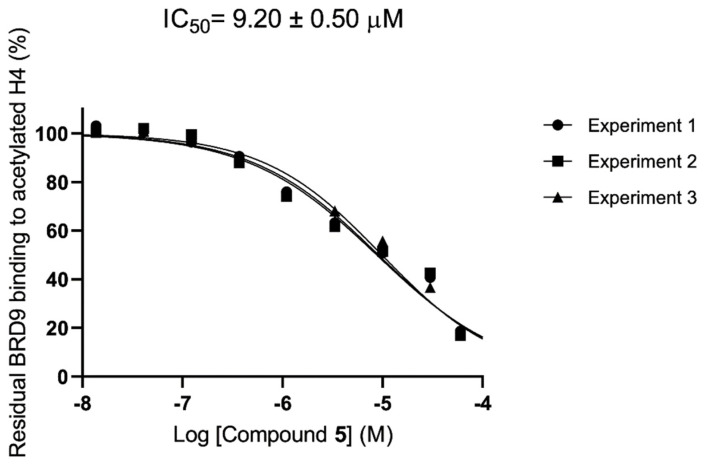
IC_50_ curve of compound **5** on BRD9. Data are presented as means with standard deviation (SD), with *n* = 3.

To address these issues and validate the importance of the molecules developed as BRD4 binders, we decided to investigate their selectivity towards two other bromodomains. Specifically, we focused on BRD3, a representative of the BET (bromo- and extra-terminal domains) family proteins, and BRD9, which belongs to the non-BET category (proteins containing bromodomains but not part of the BET family). The AlphaScreen assay revealed that none of these molecules bind to BRD3(BD1) at either 10 µM or 1 µM (Figure 6A and Table 2). On the other hand, for BRD9, only compound **5** showed binding capability but with an IC_50_ value that was twice as high compared to that for BRD4 (IC_50_ = 9.20 ± 0.50 µM) (Figure 6B and Figure 7 and Table 2). Notably, the obtained results showed the ability of the identified compounds in differently bind various bromodomains, even those within the BET family that share structural similarities. This feature is crucial for developing inhibitors that are selective and have fewer off-target effects.

## 3. Materials and Methods

### 3.1. Computational Details

#### 3.1.1. Preparation of Library

For each item of the library considered in this study, LigPrep [20] was used for the generation of all possible tautomers, stereoisomers, and protonation states at physiological pH, while QikProp [21,22,23] (Schrödinger Suite) was employed to predict pharmacokinetic parameters. After that, the output library was used for the subsequent molecular docking calculations.

#### 3.1.2. Development of 3D Structure-Based Pharmacophore Model for BRD4

In order to generate the model in the same coordinates system in which the docking experiments were performed, the crystal structure of BRD4(BD1) co-crystallized with (+)-JQ1 (PDB code: 3MXF [7]) was superimposed onto the crystal structure 4QB3 [9]. The latter was used for grid generation and as a reference protein system for both developing the pharmacophore model and performing molecular docking experiments. (+)-JQ1 co-crystallized in 3MXF was subsequently used as input for generating the “structure-based 3D pharmacophore” model through the Develop Pharmacophore Hypothesis panel, considering the “Single ligand” option. Upon manually selecting the chemical features for the pharmacophore model and considering a tolerance set to 2 Å for each feature, a seven-point pharmacophore model (“AAHHRRR”) was obtained. 

Specifically, following the definitions as implemented in the Develop Pharmacophoric Hypothesis panel (Phase [24,25,26]), “A” indicates an acceptor group, “H” a hydrophobic one, and “R” an aromatic ring. Furthermore, to validate the generated hypothesis, 35 co-crystallized BRD4 ligands were considered (2YEL, 3P5O, 3ZYU, 4F3I, 4J3I, 4LRG, 4MR3, 4O74, 4UIX, 4Z1Q, 4Z93, 5ACY, 5AD2, 5F5Z, 5F60, 5F61, 5F62, 5F63, 5FBX, 5HLS, 5I80, 5IGK, 5JWM, 5KHM, 5UOO, 5V67, 5WMD, 5Y8Y, 5YOU, 6C7R, 6CKS, 6E4A, 6Q3Y, 6Q3Z) [10,11,12,13,14,15,16,17,27,28,29,30,31,32,33,34,35,36,37,38,39,40,41,42,43,44,45]. Specifically, all the BRD4 crystal structures containing a co-crystallized inhibitor featuring a reported biological parameter and related value (e.g., IC_50_, K_D_) were downloaded and superimposed onto the reference crystal structure 4QB3 [9]. Subsequently, only the ligand structures were considered and used as input for pharmacophore screening accounting for the “score in place” option. Interestingly, 20 ligands among the starting 35 respected at least 5/7 pharmacophoric features with good PhaseScreen score values (from 1 to 2.3, Table S2).

#### 3.1.3. Ligand-Based Pharmacophore Screening

Pharmacophore screening was performed before and after molecular docking calculations. Firstly, 273 in-house synthesized compounds were preliminarily screened, accounting for the generated BRD4 pharmacophoric model (AAHHRRR model) using the “Ligand and database screening” tool in Phase [24,25,26]. Specifically, the “generate multiple conformers“ option was set, with a maximum of 50 conformers for each molecule, thus performing a conformational search aimed to evaluate the matching with the pharmacophoric features a priori. The pharmacophore screening output highlighted 158 compounds matching 5/7 pharmacophoric points, which represented the input for molecular docking experiments. 

#### 3.1.4. Preparation of Protein Structure and Molecular Docking Experiments on BRD4

The 3D protein model was prepared using the Schrödinger Protein Preparation Wizard [46], starting from the high-resolution (0.94 Å) BRD4 crystal structure (PDB code: 4QB3 [9]) co-crystallized with olinone. All hydrogens were added, and bond orders were assigned. The grid center grid had the following coordinates −24.83 × 50.20 × −2.69 and was characterized by inner and outer box dimensions of 10 × 10 × 10 and 22.55 × 22.55 × 22.55, respectively. Before performing molecular docking experiments, the re-docking of (+)-JQ1 was performed, obtaining an RMSD of 0.5 Å (Figure S3). Subsequently, molecular docking experiments of the in-house library of 273 items (Table S1, Supporting Information) were performed using Glide software (version 9.0) [46,47,48,49] and Extra Precision (XP) mode, saving a maximum number of poses of 20 for each compound for the subsequent analysis.

#### 3.1.5. Structure-Based Pharmacophore Screening

For the output docking poses of the 158 compounds, structure-based pharmacophore screening was conducted. Specifically, these items were subjected again to screening with the BRD4 pharmacophoric model, and in this case, the specific conformer accommodated in the chosen protein structure was accounted for, skipping any further conformational search (i.e., skipping the “generate multiple conformers option”, as above reported). After this latter step, only 6 of 158 molecules, i.e., **1**–**6**, matched all the pharmacophoric points, featuring a phase screen score from 0.48 to 1.35 and a docking score −3.5 kcal/mol.

### 3.2. Biological Evaluation on BRD4(BD1), BRD3(BD1), and BRD9

The ability of the selected compounds to compete with histone H4/Ac for binding to each bromodomain in a dose-response manner was assessed by AlphaScreen technology using the BRD4(BD1) Inhibitor Screening Assay Kit (BSP-32514), BRD3(BD1) Inhibitor Screening Assay Kit (BSP-32513), and BRD9 Inhibitor Screening Assay Kit (BSP-32519). The assay measured the competition between the compounds and either histone H4(1−21)K5/8/12/16Ac-BiotinOH. Starting from a stock solution of 10 mM [100% dimethyl sulfoxide (DMSO)], compounds and/or reference compounds were diluted in different assay buffers depending on the protein type. For the IC_50_ calculation, the highest compound concentration tested was 10 μM for the BRD4(BD1) and BRD3(BD1) tests, while the concentration was 60 μM for the BRD9 test, and starting from this concentration, 3-fold dilutions were prepared. A total of 2.5 μL of each dilution was transferred into a low-volume well containing 2.5 μL of protein, 2.5 μL of buffer, 1 μL of histone (50 nM in the final well), and 1.5 μL of water. The mixture was incubated for 30 min at room temperature. After splitting the mix in the plate (384-well Optiplates, PerkinElmer, Waltham, MA, USA) and adding the acceptor and the donor beads, the plates were incubated, protecting them from light at room temperature. After 60 min, the results were read on the Enspire microplate analyzer (PerkinElmer). The compound was tested in triplicate, and the data were analyzed using GraphPad Prism software (version 8.0) with the equation log(inhibitor) vs. normalized response-variable slope. The following conditions were applied for each bromodomain:

- BRD4(BD1) and BRD3(BD1): GST-tag-protein concentration = 1.6 ng/μL; buffer type = Hepes solution (BSP-31091); AlphaLISA acceptor beads = 1/250-fold dilution (PerkinElmer #AL109C), streptavidin donor beads = 1/250-fold dilution (PerkinElmer #6760002);
- BRD9: GST-tag-protein concentration = 2 ng/μL; buffer type = tris−HCl solution (BSP-33007); AlphaLISA acceptor beads = 1/250-fold dilution (PerkinElmer #AL109C), streptavidin donor beads = 1/125-fold dilution (PerkinElmer #6760002);

## 4. Conclusions

This work focused on developing a pharmacophore model specifically tailored for BRD4, ensuring high accuracy in the virtual screening process. This model was then used to scan a chemical library, pinpointing candidate molecules with the potential to bind and interfere with BRD4 activity.

The identified candidate molecules were validated using AlphaScreen assays, a highly sensitive method to confirm protein–ligand interactions and binding affinities. Three compounds showed strong potential to act as BRD4 inhibitors, with IC_50_ values between 0.60 and 4.66 µM. To investigate their selectivity profile, we tested these compounds against other bromodomains, specifically BRD3 and BRD9, and the results highlighted BRD4 selectivity.

Our findings underscore the high impact of computational techniques in modern drug discovery, demonstrating how in silico analysis can be useful in screening campaigns. Moreover, this approach provides a robust framework for developing and repurposing BRD4 inhibitors, accelerating the drug discovery process while enhancing precision and cost-effectiveness, ultimately contributing to the development of targeted treatments for diseases involving BRD4 dysregulation.

## Figures and Tables

**Table 1:** Chemical structure of compounds **1**–**6** and predicted main computational parameters.

Compound	Structure	Docking Score	PhaseScreen Score	Number of Matched Features	Interactions
**1**	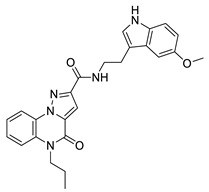	−6.6	1.1	5/7	H bond (Tyr97, Tyr139, Asn140); π-π interaction (Tyr139)
**2**	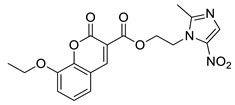	−3.7	1.2	5/7	H bond (Tyr97, Asn140)
**3**	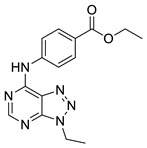	−5.7	0.5	5/7	H bond (Tyr97, Asn140, Ile146); π-π interaction (Trp81)
**4**	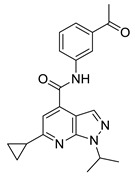	−5.1	1.2	5/7	H bond (Asn93, Tyr97, Asn140)
**5**	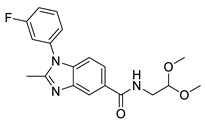	−5.2	1.0	5/7	H bond (Asn140, Lys141)
**6**	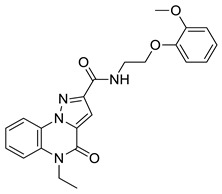	−5.9	1.3	5/7	H bond (Tyr97, Asn140, Asp144)

**Table 2:** A summary of the residual binding percentages of histone H4Ac to the selected bromodomains after treatment with compounds **1**–**6** (10 and 1 μM) and reference compounds (10 and 1 μM), along with the corresponding IC_50_ values.

Compound	Residual Binding of Histone H4Ac to BRD4(BD1) ± SD (%) [Compound] = 10 μM r	Residual Binding of Histone H4Ac to BRD4(BD1) ± SD (%) [Compound] = 1 μM r	IC_50_ ± SD (µM)	Residual Binding of Histone H4Ac to BRD3(BD1) ± SD (%) [Compound] = 10 μM r	Residual Binding of Histone H4Ac to BRD3(BD1) ± SD (%) [Compound] = 1 μM	Residual Binding of Histone H4Ac to BRD9 ± SD (%) [Compound] = 10 μM	Residual Binding of Histone H4Ac to BRD9 ± SD (%) [Compound] = 1 μM	IC_50_ ± SD (µM)
**1**	90.3 ± 4.8	98.8 ± 0.2	\	\	\	\	\	\
**2**	18.2 ± 2.9	33.5 ± 0.7	0.60 ± 0.25	94.8 ± 3.3	≥100	96.0 ± 3.7	≥100	\
**3**	72.9 ± 4.8	91.6 ± 2.3	\	\	\	\	\	\
**4**	79.6 ± 4.7	92.7 ± 2.2	\	\	\	\	\	\
**5**	37.5 ± 3.6	63.5 ± 4.9	3.46 ± 1.22	95.6 ± 4.1	≥100	52.2 ± 3.1	77.7 ± 4.7	9.20 ± 0.50
**6**	47.7 ± 0.3	63.8 ±1.3	4.66 ± 0.52	87.8 ± 8.6	≥100	91.4 ± 7.4	≥100	\
**(+)-JQ1** **(Reference compound)**	7.6 ± 1.2	30.2 ± 0.3	0.23 ± 0.03	6.1 ± 2.2	34.1 ± 2.5	\	\	\
**I-BRD9** **(Reference compound)**	\	\	\	\	\	8.6 ± 1.3	13.6 ± 0.9	2.5 ± 0.9 × 10^−2^

## Data Availability

All the relevant data are presented within the body of this paper and Supplementary Materials.

## Supplementary Materials

The following are available online at https://www.mdpi.com/article/10.3390/molecules29174025/s1, Figure S1. (A) A representation of the AAHHRRR 7-point BRD4 pharmacophore model, featuring the specified features (“A” = acceptor, “H” = hydrophobic, and “R” aromatic ring groups). (B) A representation of the AAHHRRR 7-point BRD4 pharmacophore model in the BRD4 binding site, Table S1. SMILES of the compounds belonging to the investigated in-house library. Figure S2. Docking poses of compounds **1**–**6**. Table S2. The predicted computational parameters of the 20 BRD4 known ligands respecting the generated 7-point 3D structure-based pharmacophore model “AAHHRRR”. Figure S3. The superposition of co-crystallized (+)-JQ1 (highlighted in light blue) to the reproduced docking pose of (+)-JQ1 (highlighted in violet) in the BRD4 binding site, with an RMSD value of 0.5.
