# Supplementary material for: Identification of Novel Bromodomain-Containing Protein 4 (BRD4) Binders through 3D Pharmacophore-Based Repositioning Screening Campaign
Source: Molecules. 2024 Aug 26;29(17):4025. doi: 10.3390/molecules29174025 (PMC11397543; doi:10.3390/molecules29174025)
Supplement: Supplementary file 1 [file molecules-29-04025-s001.zip › molecules-3160210-supplementary.pdf]

Identification of Novel Bromodomain-Containing Protein 4 (BRD4) Binders through 3D Pharmacophore-Based Repositioning Screening Campaign

Ester Colarusso [1,†], Erica Gazzillo [1,†], Eleonora Boccia [1], Stefania Terracciano [1], Ines Bruno [1], Giuseppe Bifulco [1], Maria Giovanna Chini [2,*] and Gianluigi Lauro [1,*]

[1] Department of Pharmacy, University of Salerno, Via Giovanni Paolo II 132, 84084 Fisciano, Italy
[2] Department of Biosciences and Territory, University of Molise, Contrada Fonte Lappone, 86090 Pesche, Italy
* Correspondence: mariagiovanna.chini@unimol.it (M.G.C.); glauro@unisa.it (G.L.); Tel.: +39-0874404132 (M.G.C.); +39-089969124 (G.L.)
† These authors contributed equally to this work.

Table of contents

Figure S1. 2

Table S1. 2

Figure S2. 10

Table S2. 10

Figure S3. 11

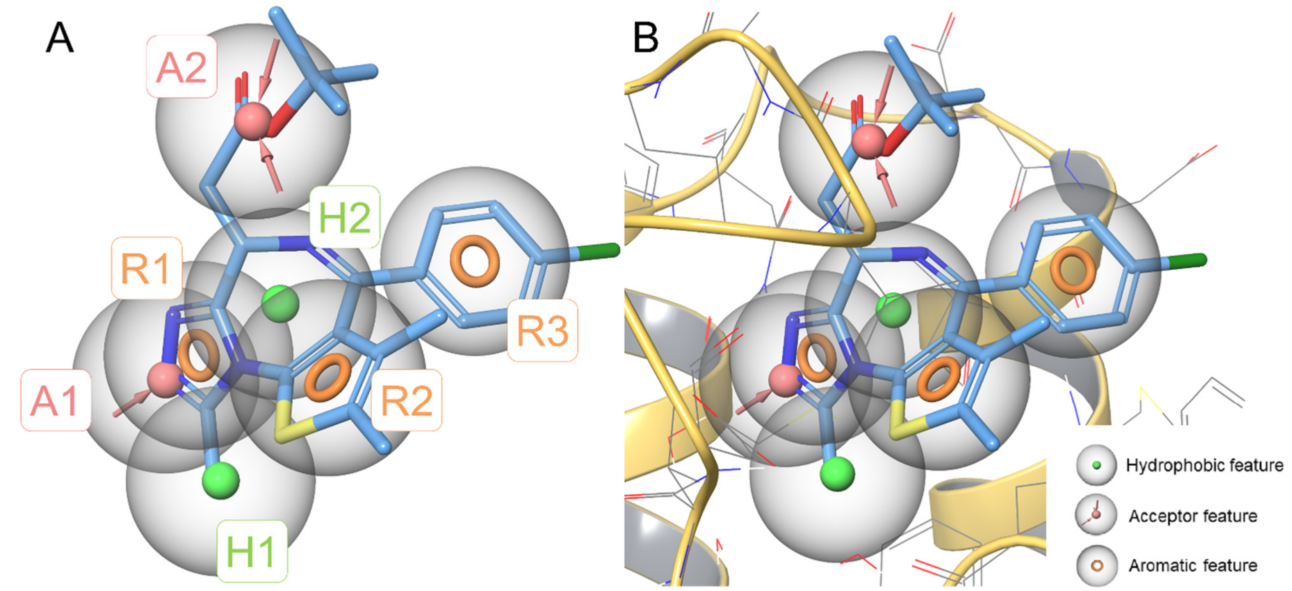

Figure S1. A) Representation of the AAHHRRR-7 point BRD4 pharmacophore model, featuring the specified features ("A" = acceptor, "H" = hydrophobic, and "R" aromatic ring groups). B) Representation of the AAHHRRR-7 point BRD4 pharmacophore model in the BRD4 binding site.

Table S1. SMILES of the compounds belonging to the investigated in-house library.

Compound	Smile
**1**	c1cccc(n(c2=O)CC)c1n(c23)nc(c3)C(=O)NCCOc4c(OC)cccc4
**2**	CCOc(ccc1)c(c12)oc(=O)c(c2)C(=O)OCCn3c([N+]([O-])=O)cnc3C
**3**	CCOC(=O)c1ccc(cc1)Nc2ncnc(c23)n(nn3)CC
**4**	CC(=O)c1cc(ccc1)NC(=O)c2cc(C3CC3)nc(c24)n(C(C)C)nc4
**5**	COC(OC)CNC(=O)c(c1)ccc(c12)n(c(n2)C)-c3cc(F)ccc3
**6**	CCCn(c1=O)c(cccc2)c2n(c13)nc(c3)C(=O)NCCc4c[nH]c(c45)ccc(c5)OC
**7**	C1CCCCc(c12)sc(c2C(=O)OCC)NC(=O)CSc(nn3)n(c34)c5c(c(C)c4)cccc5
**8**	CCOc(cc1)ccc1[C@@H]2C(C#N)=C(N)Oc3c2ccc(=O)c(c34)c(C)oc4C
**9**	CCc1nnc(s1)NC(=O)c(c2N)sc(c23)nc(C)cc3COC
**10**	O=c(o1)cc(CC)c(c2)c1c(C)c(c23)oc(C)c3C
**11**	c1cccc(c1OC)Oc(c2C)c(n[nH]2)-c3c(O)cc(O)cc3
**12**	Cc1ccc(cc1)S(=O)(=O)NCCC(=O)Oc(cc2)cc(c23)oc(=O)c(Cl)c3C
**13**	n1ccccc1NC(=O)CNC(=O)c(c2)[nH]c(c23)c(OC)ccc3OC
**14**	Cc1cc(C)cc(c12)occ2CC(=O)Nc(sc(c34)CCC4)c3C(=O)NCCCOCC
**15**	COc1ccc(OC)c(c12)[nH]c(c2)C(=O)N(C3)CCC[C@H]3c(nn4)n(c45)cccc5
**16**	COc1c(Cl)cc(cc1)NC(=O)CCNC(=O)c(c2)n(C)c(c23)cc(OC)cc3OC
**17**	Cc(n1)sc(c12)c(n[nH]c2=O)-c3cc(OC)ccc3
**18**	O=S1(=O)C[C@H](CC1)NC(=O)c(c2[O-])cnc(c23)c(Cl)ccc3
**19**	O=S1(=O)C[C@@H](CC1)NC(=O)c(c2[O-])cnc(c23)c(OC(F)(F)F)ccc3

**20**	c1ccc(OC(F)(F)F)c(c12)[nH]cc(c2=O)C(=O)N3CCN(CC3)C(=O)OCC
**21**	COc1ccc(OC)c(c12)[nH]c(c2)C(=O)NCCNC(=O)[C@H]3CCCO3
**22**	COc1ccc(OC)c(c12)[nH]c(c2)C(=O)NCCNC(=O)c3cccnc3
**23**	c1ccc(OC)c(c12)oc(c2)C(=O)Nc(c3)ccc(c34)OCO4
**24**	CCc(n1)nn(c12)c(ccn2)-c3ccc(Br)cc3
**25**	FC(F)(F)c1cc(ccc1)Nc(cc2C)nc(n2)Nc(cc3C(F)(F)F)ccc3
**26**	c1cc(F)cc(OC)c1-c(ccn2)n(c23)nc(n3)C4CCC4
**27**	c1cc(F)cc(OC)c1-c(ccn2)n(c23)nc(n3)[C@@H]4CCCO4
**28**	O=c1[nH]c(C)nc(c12)sc(c2C)C(=O)NCCc3c[nH]c(c34)ccc(Cl)c4
**29**	Cc1[nH]nc(C)c1CCC(=O)Nc(c2)ccc(c23)ncn(c3=O)C4CC4
**30**	CS(=O)(=O)N(CC1)CCN1C(=O)c(c2=O)c[nH]c(c23)c(OC)ccc3
**31**	CSCC[C@@H](CO)NC(=O)Cn(c1=O)ncc(c12)ccc(OC)c2OC
**32**	COC(=O)c1c(cccc1)NC(=O)c(c2N)sc(c23)nc(C)cc3C(F)(F)F
**33**	c1cccc(c12)n3c(n2)c(C#N)c(C)cc3-n4ccnc4
**34**	c1cccc(c12)n(c(n2)[C@@H](C)O)CCOc(cc3)ccc3C
**35**	CC(=O)c1c(=O)[nH]c2ccc(Cl)c(c2c13)C(=O)c4c3cccc4
**36**	COCCCNC(=O)c1c(sc(c12)CCCC2)NC(=O)c3cc(n[nH]3)-c(cc4)c(O)cc4C
**37**	COc(c1)ccc(OC)c1NC(=O)c2cc(n[nH]2)-c(c3C)c(O)cc(C)c3
**38**	CCNc1c(=O)n(C)c2cccc(c2c13)C(=O)c4c3cccc4
**39**	COc1c(Cl)cc(cc1)NC(=O)c(c2N)sc(c23)nc(C)cc3C
**40**	CCOc(c1)ccc(c12)ncc(C(=O)OCC)c2Nc(ccc3)cc3C(=O)OCC
**41**	CCOC(=O)c(c1)ccc(c12)nc(C)cc2Nc3c(OC)ccc(Cl)c3
**42**	CCOC(=O)c(c1)ccc(c12)nc(C)cc2Nc(c3CC)cccc3
**43**	CCOC(=O)C1=C(C)N(c2ccccc2)C(=O)N[C@@H]1c3c(Br)ccc(c3)O
**44**	CCOC(=O)C1=C(C)N(c2ccccc2)C(=O)N[C@H]1c3c(Br)ccc(c3)O
**45**	CC(=O)c1cc(ccc1)Nc(cc(n2)C)n(c23)nc(n3)-c4ccccc4
**46**	c1ccc(Cl)cc1Nc(cc(n2)C)n(c23)nc(c3)-c4cc(OC)c(cc4)OC
**47**	c1ccc(OC)cc1C(=O)N(c2ccc(Cl)cc2)Cc(n3)nn(c34)c([O-])cc(n4)C
**48**	c1cccc(F)c1Nc(cc(n2)C(C)C)n(c23)nc(n3)-c4cccnc4
**49**	o1cccc1CNC(=O)CNC(=O)c(c2)[nH]c(c23)c(OC)c(OC)c(c3)OC
**50**	FC(F)(F)c1cc(ccc1)N(C2=O)N=C(CC)\C2=C\c3c([O-])ccc(c3)[N+]([O-])=O
**51**	c1ncccc1CNC(=O)c(c2-n3cccc3)sc(c24)nc(C)cc4C
**52**	Cc(n1)ccc(cc2)c1c(c23)[nH]c(c3)C(=O)N4CCN(CC4)c5ccccn5
**53**	N#CCSc(nn1)n(c12)c3c(c(=O)n2CCC)ccc(c3)C(=O)NCC(C)C
**54**	CC(C)Cn(c(n1)CO)c(c12)cccc2
**55**	O=C1C[C@H](C#N)CN1c(c2)ccc(OC)c2OC
**56**	COc(cc1)c(OC)cc1[C@@H]2NCCc(c23)[nH+]c[nH]3
**57**	CCCN(C(=O)C1=O)c(c12)ccc(Br)c2
**58**	CCNc(n1CC)nc(c12)n(C)c(=O)[nH]c2=O
**59**	ClC1(Cl)[C@H](C1)Cn(c(n2)CO)c(c23)cccc3
**60**	CC(=O)c1cc(F)c(O)cc1
**61**	c1cc(F)ccc1-n(c2C)nc(c23)c(=O)n(C)nc3C
**62**	CC(C)c1nc(C2CC2)nc(n1)N
**63**	c1cccc2n(CCC)c(=O)c(c3c12)NC(=O)[C@H](O3)C
**64**	COCCc([nH]1)nc(c12)n(C)c(=O)n(C)c2=O
**65**	OCc(n1)[nH]c(c12)cccc2

**66**	O=c1n(C)cnc2sc(c3c12)CCCC3
**67**	CCn(c1=O)cnc2sc(c3c12)C[C@H](C)CC3
**68**	CCn(c1=O)cnc2sc(c3c12)CCCC3
**69**	CC(C)n(c1=O)cnc(c12)sc(C)c2C
**70**	CC(C)Cn(c1=O)cnc(c12)sc(c2)CC
**71**	Cc1cc(C)nc(n1)Nc2ccc(F)cc2
**72**	CNC(=O)Cc(c1C(=O)NC)n(C)cc1C
**73**	OCc(n1)n(CC)c(c12)cccc2
**74**	CCCNc(c1C)nc(=O)[nH]n1
**75**	n1[nH]c(=O)nc(c1C)N[C@@H](C)c2ccccc2
**76**	CC[C@H](C)Nc(c1C)nc(=O)[nH]n1
**77**	CCNc(c1C)nc(=O)[nH]n1
**78**	CCCNc(c1C)nc(=S)[nH]n1
**79**	n1[nH]c(=O)nc(c1C)N[C@@H]([C@@H]2C)CCCC2
**80**	Cc1c(Cl)cc(OC)c(c1)Nc(cc(n2)C)n(c23)nc(n3)-c4ccccc4
**81**	s1cc(C)n(c12)c(=O)cc(n2)C[N@@H+](CCC)Cc3cc(OC)c(OC)c(c3)OC
**82**	c1cc(F)cc(OC)c1-c(ccn2)n(c23)nc(n3)C4CCC4
**83**	O1COc(c12)ccc(c2)CNC(=O)CC3CCN(CC3)C(=O)c(c4)ccc(c45)oc(n5)C6CC6
**84**	c1cc(F)ccc1CNC(=O)CC2CCN(CC2)C(=O)c(c3)ccc(c34)oc(n4)C5CC5
**85**	CC[C@H](C)NC(=O)CSc(n1)n(CC)c(=O)c(c1c23)n(C)c2ccc(c3)OC
**86**	c1ccccc1CNC(=O)CN(C(=O)S2)C(=O)\C2=C\c3ccc(cc3)OC
**87**	n1nc(C)n(c12)c3c(cccn3)n(c2=O)CC(=O)N[C@@H](CC)c4ccc(cc4)OC
**88**	O1CCOc(c12)ccc(c2)NC(=O)CCCCCn(c3=O)c(=O)[nH]c(c34)cccc4
**89**	O1CCOc(c12)ccc(c2)NC(=O)CCCCn(c3=O)c(=O)[nH]c(c34)cccc4
**90**	c1ncc(Br)cc1NC(=O)CCCCn(c2=O)c(=O)[nH]c(c23)cccc3
**91**	c1ncc(Br)cc1NC(=O)CCCCCCn(c2=O)c(=O)[nH]c(c23)cccc3
**92**	c1nc(I)ccc1NC(=O)CCCCCn(c2=O)c(=O)[nH]c(c23)cccc3
**93**	n1csc(c12)cc(cc2)NC(=O)CCCCCn(c3=O)c(=O)[nH]c(c34)cccc4
**94**	c1cc(F)ccc1/C=C/C(NC(C)=C2C(=O)OCC)=N[C@@H]2c3c(Br)ccc(c3)O
**95**	c1cc(F)ccc1/C=C/C(NC(C)=C2C(=O)OCC)=N[C@H]2c3c(Br)ccc(c3)O
**96**	O1CCOC12CCN(CC2)C(=O)c3snc(c34)c(=O)n(c([n-]4)=O)CCC
**97**	c1cc(Cl)ccc1/C=C2/C(C)=NN(C2=O)c(cc3)ccc3[N+]([O-])=O
**98**	COc(c1)cc(OC)cc1COc(ccc2)cc2-c3nc(N)nc(c34)[nH]cn4
**99**	c1ccccc1COc(ccc2)cc2-c3nc(N)nc(c34)[nH]cn4
**100**	FC(F)(F)c1cc(c(Cl)cc1)OCc2ccc(cc2)-c3nc(N)nc(c34)[nH]cn4
**101**	COC(=O)[C@@H]1CCCN1C(=O)c(c2)nn(c23)c4c(cccc4)n(c3=O)CC(C)C
**102**	c1ccccc1C(=O)c2ccc(cc2)Cn(nn3)cc3-c4cc(O)ccc4
**103**	c1cc(O)c(O)cc1/C=C2/C(=O)N(C(=O)S2)Cc3nnc(o3)-c4ccccn4
**104**	CCOC(=O)C1=C(C)N(CCC([O-])=O)C(=O)N[C@@H]1c2ccc([N+]([O-])=O)cc2
**105**	CCOC(=O)C1=C(C)N(CCC([O-])=O)C(=O)N[C@H]1c2ccc([N+]([O-])=O)cc2
**106**	CCOC(=O)C1=C(C)N(c2ccccc2)C(=O)N[C@@H]1c3c(Br)cc(c(c3)O)OC
**107**	CCOC(=O)C1=C(C)N(c2ccccc2)C(=O)N[C@@H]1c3c(O)ccc(c3)OC
**108**	CCOC(=O)C1=C(C)N(c2ccccc2)C(=O)N[C@@H]1c3c([N+]([O-])=O)ccc([O-])c3
**109**	CCOC(=O)C1=C(C)N(c2ccccc2)C(=O)N[C@@H]1c3c([N+]([O-])=O)cccc3
**110**	CCOC(=O)C1=C(C)N(c2ccccc2)C(=O)N[C@@H]1c3cc(Br)ccc3
**111**	CCOC(=O)C1=C(C)N(c2ccccc2)C(=O)N[C@@H]1c3cc([N+]([O-])=O)ccc3

**112**	CCOC(=O)C1=C(C)N(c2ccccc2)C(=O)N[C@@H]1c3ccc(Br)cc3
**113**	CCOC(=O)C1=C(C)N(c2ccccc2)C(=O)N[C@@H]1c3ccc(Cl)cc3
**114**	CCOC(=O)C1=C(C)N(c2ccccc2)C(=O)N[C@@H]1c3ccc(O)cc3
**115**	CCOC(=O)C1=C(C)N(c2ccccc2)C(=O)N[C@@H]1c3ccc([N+]([O-])=O)cc3
**116**	CCOC(=O)C1=C(C)N(c2ccccc2)C(=O)N[C@@H]1c3ccc(cc3)OC
**117**	CCOC(=O)C1=C(C)N(c2ccccc2)C(=O)N[C@@H]1c(c3C(F)(F)F)cccc3
**118**	CCOC(=O)C1=C(C)N(c2ccccc2)C(=O)N[C@@H]1c(c(Br)cc3)cc3C(F)(F)F
**119**	CCOC(=O)C1=C(C)N(c2ccccc2)C(=O)N[C@@H]1c(c(Cl)cc3)cc3C(F)(F)F
**120**	CCOC(=O)C1=C(C)N(c2ccccc2)C(=O)N[C@@H]1c(cc3)ccc3C
**121**	CCOC(=O)C1=C(C)N(c2ccccc2)C(=O)N[C@H]1c3c(Br)cc(c(c3)O)OC
**122**	CCOC(=O)C1=C(C)N(c2ccccc2)C(=O)N[C@H]1c3c(O)ccc(c3)OC
**123**	CCOC(=O)C1=C(C)N(c2ccccc2)C(=O)N[C@H]1c3c([N+]([O-])=O)ccc([O-])c3
**124**	CCOC(=O)C1=C(C)N(c2ccccc2)C(=O)N[C@H]1c3c([N+]([O-])=O)cccc3
**125**	CCOC(=O)C1=C(C)N(c2ccccc2)C(=O)N[C@H]1c3cc(Br)ccc3
**126**	CCOC(=O)C1=C(C)N(c2ccccc2)C(=O)N[C@H]1c3cc([N+]([O-])=O)ccc3
**127**	CCOC(=O)C1=C(C)N(c2ccccc2)C(=O)N[C@H]1c3ccc(Br)cc3
**128**	CCOC(=O)C1=C(C)N(c2ccccc2)C(=O)N[C@H]1c3ccc(Cl)cc3
**129**	CCOC(=O)C1=C(C)N(c2ccccc2)C(=O)N[C@H]1c3ccc(O)cc3
**130**	CCOC(=O)C1=C(C)N(c2ccccc2)C(=O)N[C@H]1c3ccc([N+]([O-])=O)cc3
**131**	CCOC(=O)C1=C(C)N(c2ccccc2)C(=O)N[C@H]1c3ccc(cc3)OC
**132**	CCOC(=O)C1=C(C)N(c2ccccc2)C(=O)N[C@H]1c(c3C(F)(F)F)cccc3
**133**	CCOC(=O)C1=C(C)N(c2ccccc2)C(=O)N[C@H]1c(c(Br)cc3)cc3C(F)(F)F
**134**	CCOC(=O)C1=C(C)N(c2ccccc2)C(=O)N[C@H]1c(c(Cl)cc3)cc3C(F)(F)F
**135**	CCOC(=O)C1=C(C)N(c2ccccc2)C(=O)N[C@H]1c(cc3)ccc3C
**136**	[O-]C(=O)c1cc(ccc1)[C@@H]2NC(=O)N(C(C)=C2C(=O)OCC)c3ccccc3
**137**	[O-]C(=O)c1cc(ccc1)[C@H]2NC(=O)N(C(C)=C2C(=O)OCC)c3ccccc3
**138**	COc(c(c1)O)cc(Br)c1/C=C2/C(C)=NN(C2=O)c(cc3)ccc3[N+]([O-])=O
**139**	FC(F)(F)c1cc(c(Cl)cc1)/C=C2/C(C)=NN(C2=O)c(cc3)ccc3[N+]([O-])=O
**140**	N#Cc1cc(ccc1)-c(n2)cccc2/C=C3/C(C)=NN(C3=O)c(cc4)ccc4[N+]([O-])=O
**141**	[O-]C(=O)c1cc(ccc1)/C=C2/C(C)=NN(C2=O)c(cc3)ccc3[N+]([O-])=O
**142**	c1c(O)ccc(Br)c1/C=C2/C(C)=NN(C2=O)c(cc3)ccc3[N+]([O-])=O
**143**	CCOc(ccc1)c([O-])c1/C=C2/C(C(C)C)=NN(C2=O)c3cnccn3
**144**	CCOc(ccc1)c([O-])c1/C=C2/C(CC)=NN(C2=O)c3c(F)cc(F)cc3
**145**	CCOc(ccc1)c([O-])c1/C=C2/C(CC)=NN(C2=O)c3cnccn3
**146**	FC(F)(F)c1cc(ccc1)-c(o2)ccc2/C=C3/C(CC)=NN(C3=O)c(cc4C(F)(F)F)ccc4
**147**	FC(F)(F)c1cc(ccc1)N(C2=O)N=C(C(C)C)\C2=C\c3cc([O-])c([N+]([O-])=O)cc3
**148**	FC(F)(F)c1cc(ccc1)N(C2=O)N=C(CC)\C2=C\c3c([O-])c(ccc3)OCC
**149**	N#Cc1ccc(cc1)N(C2=O)N=C(C)\C2=C\c3c([O-])ccc(c3)[N+]([O-])=O
**150**	[O-][N+](=O)c(c1)ccc([O-])c1/C=C2/C(C)=NN(C2=O)c3c(F)cc(Br)cc3
**151**	[O-][N+](=O)c(c1)ccc([O-])c1/C=C2/C(C)=NN(C2=O)c(cc3[N+]([O-])=O)ccc3
**152**	[O-][N+](=O)c(c1)ccc([O-])c1/C=C2/C(CC)=NN(C2=O)c3c(F)cc(F)cc3
**153**	[O-][N+](=O)c(c1)ccc([O-])c1/C=C2/C(CC)=NN(C2=O)c3cnccn3
**154**	c1ccc(OC)c([O-])c1/C=C2/C(CC)=NN(C2=O)c3c(F)cc(F)cc3
**155**	c1ccc(OC)c([O-])c1/C=C2/C(CC)=NN(C2=O)c3cnccn3
**156**	n1ccncc1N(C2=O)N=C(C)\C2=C\c3cc(ccc3)Oc4ccccc4
**157**	CCOC(=O)CN(C(=O)S1)C(=O)\C1=C\c2c([O-])ccc(c2)[N+]([O-])=O

**158**	CCOC(=O)CN(C(=O)S1)C(=O)\C1=C\c2cc(O)c(O)cc2
**159**	CCOC(=O)CN(C(=O)S1)C(=O)\C1=C\c2cc(OCc3ccccc3)c(cc2)OCc4ccccc4
**160**	c1ccccc1OC(=O)CN(C(=O)S2)C(=O)\C2=C\c3cc([O-])c([N+]([O-])=O)cc3
**161**	c1cc(F)ccc1/C=C/C(N=C(C)/C2=C(\[O-])OCC)=[NH+][C@@H]2c3ccc(cc3)OCC=C
**162**	c1cc(F)ccc1/C=C/C(N=C(C)/C2=C(\[O-])OCC)=[NH+][C@H]2c3ccc(cc3)OCC=C
**163**	c1cc([N+]([O-])=O)ccc1[C@@H]2[NH+]=C(N=C(C)/C2=C(\[O-])OCC)\C=C\c3ccc(F)cc3
**164**	c1cc([N+]([O-])=O)ccc1[C@H]2[NH+]=C(N=C(C)/C2=C(\[O-])OCC)\C=C\c3ccc(F)cc3
**165**	c1cc(Cl)ccc1C(=O)NC(S2)=N[C@@H](c3ccc(F)cc3)C=C2c4ccccc4
**166**	c1cc(Cl)ccc1C(=O)NC(S2)=N[C@@H](c3ccccc3)C=C2c4ccccc4
**167**	c1cc(Cl)ccc1C(=O)NC(S2)=N[C@H](c3ccc(F)cc3)C=C2c4ccccc4
**168**	c1cc(Cl)ccc1C(=O)NC(S2)=N[C@H](c3ccccc3)C=C2c4ccccc4
**169**	c1ccccc1C2=C[C@@H](N=C(S2)N)c3ccc(F)cc3
**170**	c1ccccc1C2=C[C@H](N=C(S2)N)c3ccc(F)cc3
**171**	c1ccccc1C(=O)NC(S2)=N[C@@H](c3ccc(F)cc3)C=C2c4ccccc4
**172**	c1ccccc1C(=O)NC(S2)=N[C@@H](c3ccccc3)C=C2c4ccccc4
**173**	c1ccccc1C(=O)NC(S2)=N[C@H](c3ccc(F)cc3)C=C2c4ccccc4
**174**	c1ccccc1C(=O)NC(S2)=N[C@H](c3ccccc3)C=C2c4ccccc4
**175**	COc(cc1)ccc1C(=O)NC(S2)=N[C@@H](c3ccccc3)C=C2c4ccccc4
**176**	COc(cc1)ccc1C(=O)NC(S2)=N[C@H](c3ccccc3)C=C2c4ccccc4
**177**	c1cc(C)ccc1C(=O)NC(S2)=N[C@@H](c3ccccc3)C=C2c4ccccc4
**178**	c1cc(C)ccc1C(=O)NC(S2)=N[C@H](c3ccccc3)C=C2c4ccccc4
**179**	c1cc(F)ccc1C(=O)NC(S2)=N[C@@H](c3ccccc3)C=C2c4ccccc4
**180**	c1cc(F)ccc1C(=O)NC(S2)=N[C@H](c3ccccc3)C=C2c4ccccc4
**181**	c1cc([N+]([O-])=O)ccc1C(=O)NC(S2)=N[C@@H](c3ccccc3)C=C2c4ccccc4
**182**	c1cc([N+]([O-])=O)ccc1C(=O)NC(S2)=N[C@H](c3ccccc3)C=C2c4ccccc4
**183**	COc(cc1)ccc1C(=O)NC(S2)=N[C@@H](c3ccc(F)cc3)C=C2c4ccccc4
**184**	COc(cc1)ccc1C(=O)NC(S2)=N[C@H](c3ccc(F)cc3)C=C2c4ccccc4
**185**	c1cc(C)ccc1C(=O)NC(S2)=N[C@@H](c3ccc(F)cc3)C=C2c4ccccc4
**186**	c1cc(C)ccc1C(=O)NC(S2)=N[C@H](c3ccc(F)cc3)C=C2c4ccccc4
**187**	c1cc(F)ccc1C(=O)NC(S2)=N[C@@H](c3ccc(F)cc3)C=C2c4ccccc4
**188**	c1cc(F)ccc1C(=O)NC(S2)=N[C@H](c3ccc(F)cc3)C=C2c4ccccc4
**189**	c1cc([N+]([O-])=O)ccc1C(=O)NC(S2)=N[C@@H](c3ccc(F)cc3)C=C2c4ccccc4
**190**	c1cc([N+]([O-])=O)ccc1C(=O)NC(S2)=N[C@H](c3ccc(F)cc3)C=C2c4ccccc4
**191**	FC(F)(F)c1cc(c(Cl)cc1)OCc2cc(ccc2)-c3nc(N)nc(c34)[nH]cn4
**192**	c1cc(C)cc(c1C(C)C)OCc2ccc(cc2)-c3nc(N)nc(c34)[nH]cn4
**193**	c1ccc(Cl)cc1COc(ccc2)cc2-c3nc(N)nc(c34)[nH]cn4
**194**	c1ccc(F)cc1COc(ccc2)cc2-c3nc(N)nc(c34)[nH]cn4
**195**	c1ccc(OC)cc1COc(ccc2)cc2-c3nc(N)nc(c34)[nH]cn4
**196**	c1cccc(c12)cccc2OCc3cc(ccc3)-c4nc(N)nc(c45)[nH]cn5
**197**	c1ccccc1COc(c2C)cc(cc2)-c3nc(N)nc(c34)[nH]cn4
**198**	n1c[nH]c(c12)nc(N)nc2-c3cc(ccc3)Oc4ccccc4
**199**	CC(C)Oc(cc1)ccc1-c(cc2)ccc2-c3nc(N)nc(c34)[nH]cn4
**200**	COc1c(OC)cc(cc1OC)-c2nc(N)nc(c23)[nH]cn3
**201**	FC(F)(F)Oc1ccc(cc1)OCc2cc(ccc2)-c3nc(N)nc(c34)[nH]cn4
**202**	FC(F)(F)c1cc(ccc1)COc(ccc2)cc2-c3nc(N)nc(c34)[nH]cn4
**203**	c1cc(F)ccc1COc(ccc2)cc2-c3nc(N)nc(c34)[nH]cn4

**204**	c1ccccc1COc(c2C)cc(cc2)-c3nc(Cl)nc(c34)[nH]cn4
**205**	n1c[nH]c(c12)nc(N)nc2-c(cc3C(F)(F)F)cc(c3)OCc4ccc(cc4)OC
**206**	n1c[nH]c(c12)nc(N)nc2-c3ccc(I)cc3
**207**	O1COc(c12)ccc(c2)C(=O)OCc3c(C)n(nn3)-c4nccs4
**208**	O1COc(c12)ccc(c2)C(=O)OCc3cn(nn3)-c(cc4)cc(c45)scn5
**209**	O1COc(c12)ccc(c2)C(=O)OCc3cn(nn3)Cc4nc(no4)-c5cccnc5
**210**	O1COc(c12)ccc(c2)C(=O)OCc3cn(nn3)Cc4nnc(o4)-c5cccnc5
**211**	c1cc(O)cc(O)c1C(=O)OCc2c(C)n(nn2)-c3nccs3
**212**	c1ccc(OC)cc1C(=O)OCc2c(C)n(nn2)-c3nccs3
**213**	c1ccc(OC)cc1C(=O)OCc2cn(nn2)-c(cc3)cc(c34)scn4
**214**	c1ccc([N+]([O-])=O)cc1C(=O)OCc2c(C)n(nn2)-c3nccs3
**215**	c1ccc([N+]([O-])=O)cc1C(=O)OCc2cn(nn2)-c(cc3)cc(c34)scn4
**216**	c1ccc([N+]([O-])=O)cc1C(=O)OCc2cn(nn2)C[C@@H](O3)COc(c34)cccc4
**217**	c1ccc([N+]([O-])=O)cc1C(=O)OCc2cn(nn2)C[C@H](O3)COc(c34)cccc4
**218**	c1ccc([N+]([O-])=O)cc1C(=O)OCc2cn(nn2)Cc3ccc(cc3)-c4ccccc4
**219**	c1ccc([N+]([O-])=O)cc1C(=O)OCc2cn(nn2)Cc3nc(no3)-c4cccnc4
**220**	c1ccc([N+]([O-])=O)cc1C(=O)OCc2cn(nn2)Cc3nnc(o3)-c4cccnc4
**221**	c1cccc(c12)nccc2C(=O)OCc3cn(nn3)-c(cc4)cc(c45)scn5
**222**	c1ccccc1C(=O)c2ccc(cc2)Cn(nn3)cc3COC(=O)c4cc([N+]([O-])=O)ccc4
**223**	c1ccccc1C(=O)c2ccc(cc2)Cn(nn3)cc3COC(=O)c(c4)ccc(c45)OCO5
**224**	c1cnccc1C(=O)OCc2c(C)n(nn2)-c3nccs3
**225**	FC(F)(F)c1cc(ccc1)CCn(nn2)cc2-c3cc(O)ccc3
**226**	FC(F)(F)c1cc(ccc1)CCn(nn2)cc2-c3ccccn3
**227**	[O-]C(=O)c1cc([N+]([O-])=O)c(cc1)Cn(nn2)cc2-c3cc(O)ccc3
**228**	[O-]C(=O)c1cc([N+]([O-])=O)c(cc1)Cn(nn2)cc2-c3ccccn3
**229**	c1ccc(O)cc1-c2cn(nn2)Cc3ccc(cc3)-c4ccccc4
**230**	c1ccc(O)cc1-c2cn(nn2)Cc3nnc(o3)-c4cccnc4
**231**	c1cccc(c12)OC[C@@H](O2)Cn(nn3)cc3-c4cc(O)ccc4
**232**	c1cccc(c12)OC[C@@H](O2)Cn(nn3)cc3-c4ccccn4
**233**	c1cccc(c12)OC[C@H](O2)Cn(nn3)cc3-c4cc(O)ccc4
**234**	c1cccc(c12)OC[C@H](O2)Cn(nn3)cc3-c4ccccn4
**235**	c1cccc(c12)[nH]cc2CCn(nn3)cc3-c4cc(O)ccc4
**236**	c1cccc(c12)[nH]cc2CCn(nn3)cc3-c4ccccn4
**237**	c1ccccc1C(=O)c2ccc(cc2)Cn(nn3)cc3-c4ccccn4
**238**	c1ncccc1-c(no2)nc2Cn(nn3)cc3-c4cc(O)ccc4
**239**	n1ccccc1-c2cn(nn2)Cc3ccc(cc3)-c4ccccc4
**240**	n1ccccc1-c2cn(nn2)Cc3nc(no3)-c4cccnc4
**241**	n1ccccc1-c2cn(nn2)Cc3nnc(o3)-c4cccnc4
**242**	FC(F)(F)c1cc(ccc1)NC(=O)CN(C(=O)S2)C(=O)\C2=C\c3c([O-])cc(OC)cc3OC
**243**	[O-][N+](=O)c1c(Cl)ccc(c1)NC(=O)CN(C(=O)S2)C(=O)\C2=C\c(n3)ccc(c34)cccc4[O-]
**244**	c1cc(O)c(O)cc1/C=C2/C(=O)N(C(=O)S2)CCc3c[nH]c(c34)cccc4
**245**	c1ccc(Cl)c([O-])c1/C=C2/C(=O)N(C(=O)S2)Cc3nc(no3)-c4ccccn4
**246**	c1ccc(F)cc1CCNC(=O)CN(C(=O)S2)C(=O)\C2=C\c3c([O-])c([N+]([O-])=O)cc(c3)OC
**247**	c1ccc(O)c(Cl)c1/C=C2/C(=O)N(C(=O)S2)Cc3nnc(o3)-c4ccccn4
**248**	c1ccccc1OC(=O)CN(C(=O)S2)C(=O)\C2=C\c3c([O-])c([N+]([O-])=O)cc(Br)c3
**249**	c1ccccc1OC(=O)CN(C(=O)S2)C(=O)\C2=C\c3cc(O)c(O)cc3

**250**	CCOC(=O)C1=C(C)N(CCC([O-])=O)C(=O)N[C@@H]1c2ccccn2
**251**	CCOC(=O)C1=C(C)N(CCC([O-])=O)C(=O)N[C@@H]1c(c2=O)coc(c23)ccc(Br)c3
**252**	CCOC(=O)C1=C(C)N(CCC([O-])=O)C(=O)N[C@@H]1c(cc2C([O-])=O)ccc2
**253**	CCOC(=O)C1=C(C)N(CCC([O-])=O)C(=O)N[C@@H]1c(n2)cccc2-c(ccc3)cc3C(=O)OC
**254**	CCOC(=O)C1=C(C)N(CCC([O-])=O)C(=O)N[C@H]1c2ccccn2
**255**	CCOC(=O)C1=C(C)N(CCC([O-])=O)C(=O)N[C@H]1c(c2=O)coc(c23)ccc(Br)c3
**256**	CCOC(=O)C1=C(C)N(CCC([O-])=O)C(=O)N[C@H]1c(cc2C([O-])=O)ccc2
**257**	CCOC(=O)C1=C(C)N(CCC([O-])=O)C(=O)N[C@H]1c(n2)cccc2-c(ccc3)cc3C(=O)OC
**258**	CCOC(=O)C1=C(C)NC(=S)N[C@@H]1c2cc(ccc2)OCC
**259**	CCOC(=O)C1=C(C)NC(=S)N[C@H]1c2cc(ccc2)OCC
**260**	COc(cc1)ccc1/C=C/C(N=C(C)\C2=C(\[O-])OCC)=[NH+][C@@H]2c3cc(ccc3)OCC
**261**	COc(cc1)ccc1/C=C/C(N=C(C)\C2=C(\[O-])OCC)=[NH+][C@H]2c3cc(ccc3)OCC
**262**	FC(F)(F)c1ccc(cc1)/C=C/C(N=C(C)/C2=C(\[O-])OCC)=[NH+][C@@H]2c3cc(ccc3)OCC
**263**	FC(F)(F)c1ccc(cc1)/C=C/C(N=C(C)/C2=C(\[O-])OCC)=[NH+][C@H]2c3cc(ccc3)OCC
**264**	c1cc(Cl)ccc1/C=C/C(N=C(C)/C2=C(\[O-])OCC)=[NH+][C@@H]2c3cc(ccc3)OCC
**265**	c1cc(Cl)ccc1/C=C/C(N=C(C)/C2=C(\[O-])OCC)=[NH+][C@H]2c3cc(ccc3)OCC
**266**	c1cc(F)ccc1/C=C/C(N=C(C)/C2=C(\[O-])OCC)=[NH+][C@@H]2c3cc(ccc3)OCC
**267**	c1cc(F)ccc1/C=C/C(N=C(C)/C2=C(\[O-])OCC)=[NH+][C@H]2c3cc(ccc3)OCC
**268**	c1ccccc1/C=C/C(N=C(C)/C2=C(\[O-])OCC)=[NH+][C@@H]2c3cc(ccc3)OCC
**269**	c1ccccc1/C=C/C(N=C(C)/C2=C(\[O-])OCC)=[NH+][C@H]2c3cc(ccc3)OCC
**270**	c1cc(C)ccc1/C=C/C(N=C(C)/C2=C(\[O-])OCC)=[NH+][C@@H]2c3cc(ccc3)OCC
**271**	c1cc(C)ccc1/C=C/C(N=C(C)/C2=C(\[O-])OCC)=[NH+][C@H]2c3cc(ccc3)OCC
**272**	c1ccccc1C2=C[C@@H](N=C(S2)N)c3ccccc3
**273**	c1ccccc1C2=C[C@H](N=C(S2)N)c3ccccc3

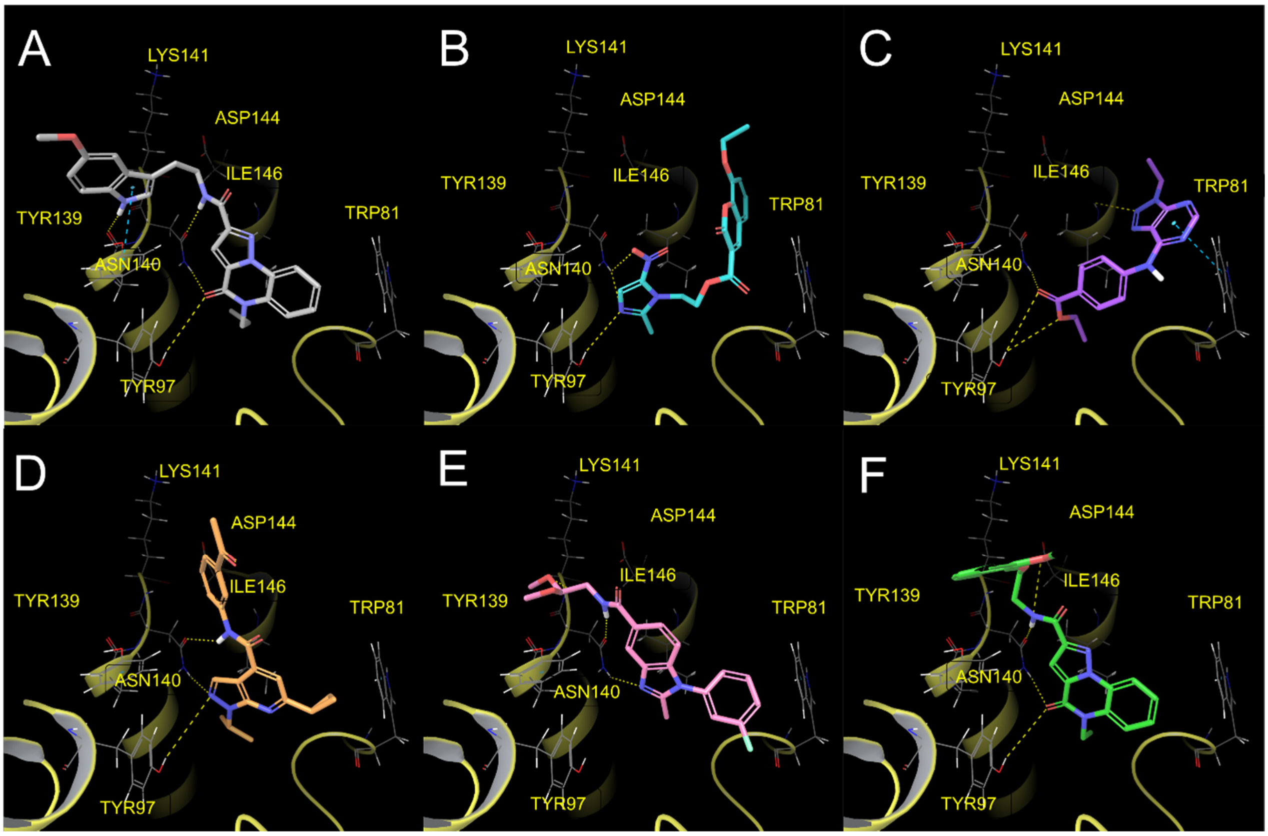

Figure S2. Docking poses of compounds **1** (panel A, colored by atom type: C grey, O red, N blue, S yellow, polar H light grey), **2** (panel B, colored by atom type: C torquoise, O red, N blue, S yellow, polar H light grey), **3** (panel C, colored by atom type: C violet, O red, N blue, S yellow, polar H light grey), **4** (panel D, colored by atom type: C orange, O red, N blue, S yellow, polar H light grey), **5** (panel E, colored by atom type: C pink, O red, N blue, S yellow, polar H light grey), and **6** (panel F, colored by atom type: C green, O red, N blue, S yellow, polar H light grey) in BRD4 binding site (PDB code: 4QB3). H-bonds and π-π interactions are reported in yellow and cyan, respectively.

Table S2. Predicted computational parameters of the 20 BRD4 known ligands respecting the generated 7-point 3D structure-based pharmacophore model "AAHHRRR".

PDB compound code	Number of matched features	PhaseScreen score
2YEL	7	2.299
3P5O	7	2.401
3ZYU	5	1.433
4F3I	7	2.667
4LRG	7	2.335
4Z1Q	6	2.026
4Z93	5	1.347
5ACY	5	1.433
5FBX	5	1.663
5HLS	7	2.367
5IGK	6	1.599
5JWM	7	2.143
5UOO	5	1.249
5V67	5	1.204
5WMD	7	2.41
5Y8Y	5	1.269
5YOU	5	1.068
6C7R	5	1.502
6Q3Y	5	1.295
6Q3Z	5	1.411

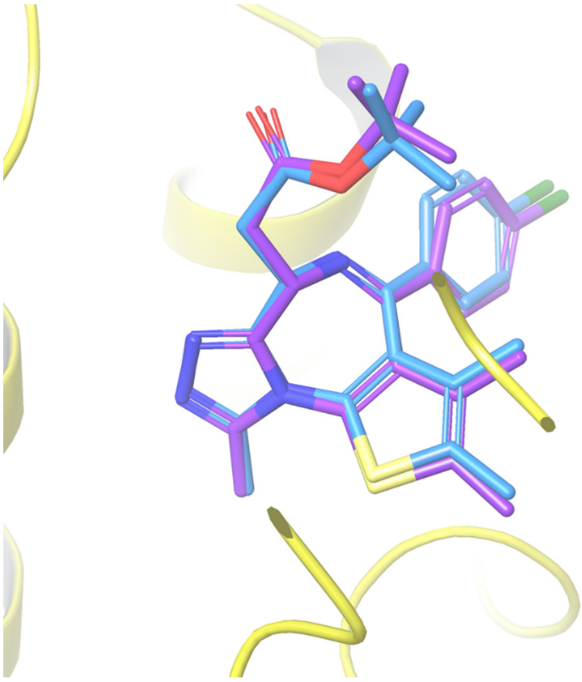

Figure S3. Superposition of co-crystallized (+)-JQ1 (highlighted in light blue) to the reproduced docking pose of (+)-JQ1 (highlighted in violet) in the BRD4 binding site, with an RMSD value of 0.5.
